# Diagnostic efficacy of multimodal imaging in non-mass-like breast lesions and correlation with pathological findings

**DOI:** 10.3389/fonc.2025.1719528

**Published:** 2025-12-12

**Authors:** Yuhan Wang, Xiuliang Wei, Mei Wu

**Affiliations:** 1 The Second Clinical College,Shandong University, Jinan, Shandong, China; 2 Department of Ultrasound, The Second Qilu Hospital of Shandong University, Jinan, Shandong, China

**Keywords:** BI-RADS, breast non-mass-like lesions, ultrasonography, digital mammography, breast magnetic resonance dynamic enhancement scanning

## Abstract

**Objective:**

This study aimed to identify key imaging features related to malignancy in Non-mass breast lesions (NMLs) via ultrasonography, systematically evaluate the diagnostic performance of ultrasound (US), digital mammography (DM), and dynamic contrast-enhanced magnetic resonance imaging (DCE-MRI). Additionally, it assessed the diagnostic independence and complementarity of these modalities within a multimodal framework to inform individualized imaging strategies.

**Measure:**

This retrospective study included female patients who underwent breast US between January 2017 and March 2025. Imaging data (including US, DM, and DCE-MRI) and clinicopathological records were collected. Chi-square and Fisher’s exact tests were used to analyze the association between US features and pathological outcomes, while multivariate binary logistic regression was employed to construct a predictive model. Receiver operating characteristic (ROC) curves were plotted, and the area under the curve (AUC) was calculated for each modality. Lastly, DeLong’s test was used to compare diagnostic efficacy, and the clinical added value of multimodal diagnosis was evaluated.

**Results:**

A total of 235 female patients (mean age, 48.06 ± 12.46 years) with 240 ultrasound-defined non-mass-like breast lesions were included, comprising 115 benign (47.9%) and 125 malignant (52.1%) cases. Among the clinical and ultrasound manifestations, age ≥45 years, palpable mass, lesion diameter ≥1.5 cm, abundant blood flow and microcalcifications, structural distortion, and abnormal axillary lymph nodes demonstrated a strong correlation with the risk of malignancy of non-massive lesions of the breast in both univariate and multivariate analyses. Significant differences in diagnostic performance metrics (accuracy, sensitivity, specificity, positive predictive value) were noted among US, DM, and DCE-MRI (all *P*<0.05). Although combined multimodal approaches (serial or parallel) improved certain diagnostic parameters (e.g., sensitivity or specificity, *P*<0.05), their AUC values were non-inferior to individual modalities (*P*>0.05). Finally, US had a significantly higher AUC than DM (*P*=0.0155), while DCE-MRI and combined methods did not outperform US alone (all *P*>0.05).

**Conclusion:**

US has demonstrated diagnostic efficacy comparable to MRI and superior to DM in the initial assessment of NML. A refined classification system for NML based on imaging-pathology correspondence should be developed in the future. US showed balanced diagnostic performance, comparable to DCE-MRI and superior to DM for initial evaluation of non-mass-like breast lesions. A refined classification system for NMLs based on imaging-pathology correspondence should be developed in the future.

## 1 Introduction

As is well documented, breast cancer remains one of the most prevalent malignancies in females worldwide. Although the 5-year survival rates have improved in recent years (1), breast cancer still accounts for approximately one-sixth of cancer-related deaths in women globally (2). Early screening and diagnosis can reduce mortality rates and enhance survival and quality of life (3). Accordingly, achieving accurate and timely diagnosis is critical for optimizing disease management and patient outcomes. At present, primary imaging modalities for the clinical screening and diagnosis of breast diseases include ultrasonography (US), digital mammography (DM), and dynamic contrast-enhanced magnetic resonance imaging (DCE-MRI, MRI). US is widely used owing to its real-time imaging capability and lack of ionizing radiation (4–6). Breast lesions are generally classified on ultrasound as mass lesions (MLs) or non-mass-like lesions (NMLs). Based on the presence or absence of a distinct space-occupying effect. MLs typically exhibit well-defined borders and are associated with posterior acoustic features such as enhancement or attenuation (7). In contrast, NML is defined as a focal area of hypoechoic change that lacks a significant occupying effect in at least two mutually perpendicular views, typically blending with surrounding breast tissue and retaining normal architectural features (11–13). Compared with mass lesions, NML exhibits more diverse morphological characteristics and frequently lacks clear boundaries, which poses challenges in accurate diagnosis. Notably, NML encompasses a wide range of pathological types, with a malignancy rate of approximately 35%-55% (14–16). Therefore, early identification and accurate stratification of NMLs are essential for guiding clinical management and treatment strategies.

This study aimed to investigate the diagnostic efficacy of three imaging methods (US, DM, and MRI) in the diagnosis of NML by systematically evaluating their effectiveness. US imaging features of NML were retrospectively analyzed, and their correlation with pathological findings was examined to increase the detection rate of ultrasound for malignant breast NML, thereby enabling early and precise intervention.

## 2 Materials and methods

### 2.1 Patients and lesions

The imaging data of patients who underwent breast ultrasonography at the Second Hospital of Shandong University from January 2017 to March 2025 were retrospectively analyzed, and a total of 240 NML lesions were screened in 235 female patients (mean age 48.06 ± 12.46 years, age range 14–79 years).

All patients underwent standardized breast ultrasound examination with complete clinical data and met the inclusion and exclusion criteria of the study. The inclusion criteria were as follows: 1. Ultrasound images meeting the definition of NML 2. Breast ultrasound examination performed at our hospital prior to treatments and complete imaging data 3. Available clinical and medical records 4. Breast biopsy or surgical resection (including minimally invasive excision of breast masses and total mastectomy) performed in our hospital, with a clear pathological diagnosis. The exclusion criteria were as follows: 1. Unclear breast ultrasound images or incomplete imaging data; 2. Ultrasound images showing typical mass lesions or lesions with both mass-type and non-mass-type manifestations; 3. Lack of clear pathological diagnosis; 4. Receipt of relevant treatments (surgery, puncture, chemotherapy, etc.) prior to imaging examination; 5. Interval of more than one month between ultrasound examination and surgery or biopsy.

**Figure 1:**
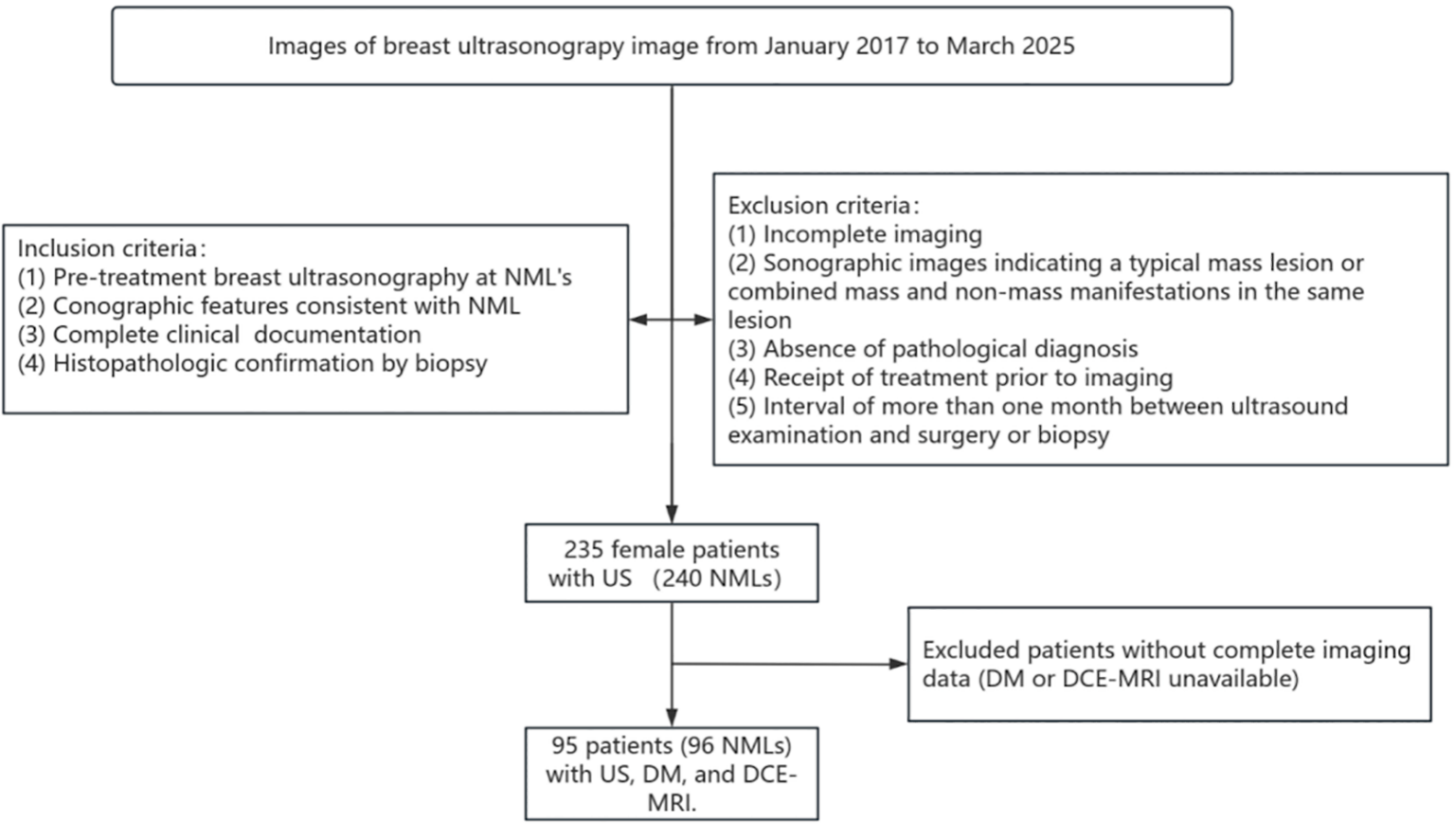
Screening flowchart.

The DM and MRI findings of patients were then retrospectively collected. A specific study flowchart is illustrated in (**Figure 1**).

### 2.2 Definition of mass lesions and non-mass-like lesions

ML is described as a three-dimensional space-occupying lesion that is apparent on two separate projections and has a distinct shape, margin, and internal echo pattern in the ACR BI-RADS^®^ Atlas (5th edition, 2013).

NML, on the other hand, is an area with changed echotexture or enhancement that lacks a distinct mass contour or space-occupying impact. It frequently exhibits regional alterations, architectural distortion, or ductal distribution.

### 2.3 Collection of pathologic findings

All included patients underwent either ultrasound-guided core needle biopsy (n = 72) or surgical excision (n = 168) at our institution. For patients who underwent both biopsy and surgical treatment, the final diagnosis was based on pathological findings.

### 2.4 Image acquisition and interpretation

Ultrasound examinations were performed using high-frequency linear array transducers (7.5–10 MHz) across the three systems: LOGIQ E9/Fortis (GE Healthcare, Chicago, IL, USA), Resona 7S, and Eagus R9S (Mindray, Shenzhen, China). All lesions were assessed in both radial and anti-radial scanning planes through real-time scanning.

**Figure 2:**
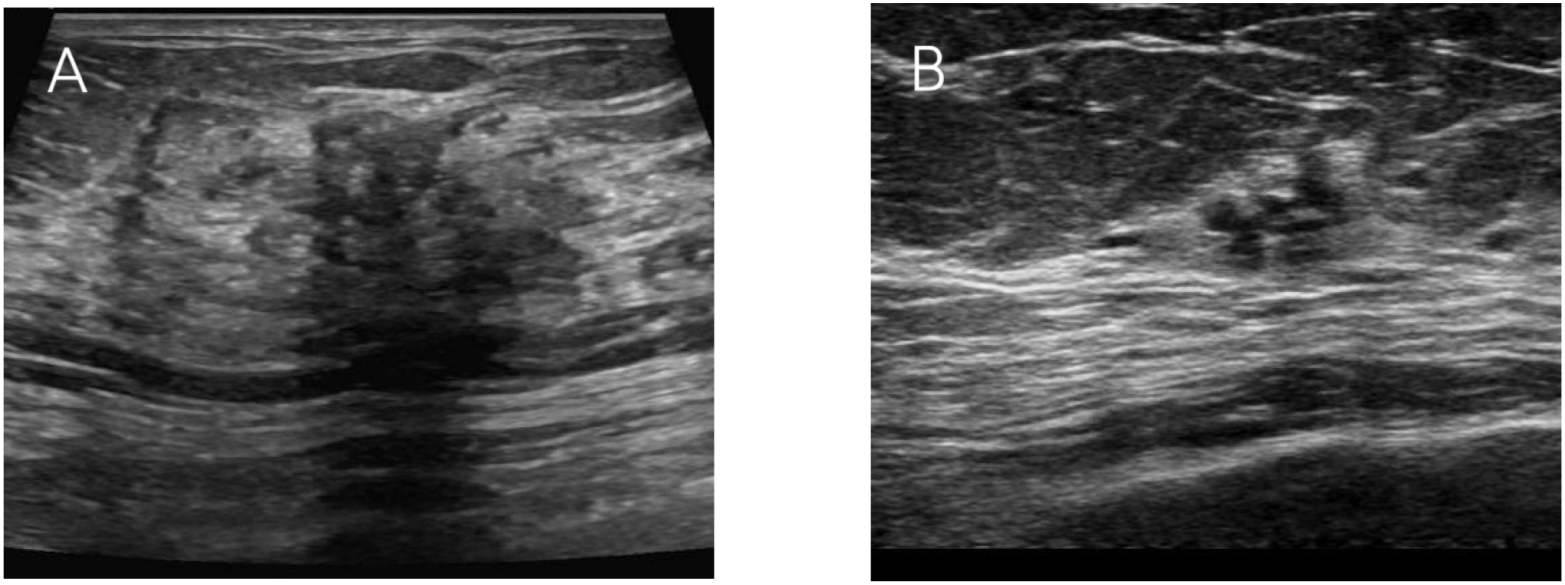
**(A)** Representative image of a typical posterior acoustic shadow. **(B)** Distortion of the glandular structure with visible lesion retraction.

Ultrasound features collected comprised internal calcification, architectural distortion, maximum lesion diameter, posterior echogenicity, color Doppler flow imaging (CDFI) signals, and abnormal axillary lymph nodes. Architectural distortion was defined as alterations in the normal breast architecture without a distinct mass, characterized by focal retraction, tethering of surrounding tissues, or radiating spiculations from a central point, in the absence of a space-occupying lesion (**Figure 2**). Abnormal axillary lymph nodes were identified based on the following criteria: loss or displacement of lymph node hilum, irregular margins, uneven or hypoechoic internal echogenicity, an aspect ratio <1.5, cortical thickness >3 mm, or eccentric cortical thickening.

DM images were acquired using a full-field digital mammography system (Selenia Dimensions; Hologic, Marlborough, MA, USA). The images covered bilateral craniocaudal (CC) and mediolateral oblique (MLO) views of the breasts.

DCE-MRI was performed using two 3.0-T scanners (Discovery MR750w and Signa Pioneer; GE Healthcare, Milwaukee, WI, USA). Multiple post-contrast dynamic series were acquired to evaluate lesion enhancement characteristics, including time-signal intensity curves and early enhancement patterns.

All imaging data were acquired using standardized protocols and retrospectively reviewed. Image interpretation was independently performed by two radiologists, each with over 10 years of experience in breast imaging. Lesions were evaluated according to the American College of Radiology Breast Imaging Reporting and Data System (BI-RADS), using a double-blinded assessment protocol. In cases of disagreement, the radiologists jointly re-evaluated the images to reach a consensus. If consensus could not be achieved, a third senior radiologist with over 20 years of diagnostic experience adjudicated the final classification. Diagnostic performance metrics, including accuracy, sensitivity, specificity, positive predictive value (PPV), and negative predictive value (NPV), were calculated for US, DM, and MRI. In addition, a parallel comparative analysis was conducted to evaluate the diagnostic contribution of each modality individually and in multimodal combinations.

### 2.5 Data analysis

Statistical analyses were performed using SPSS 29.0.1.0 and Python 3.10. Normally distributed continuous variables were presented as mean ± standard deviation. Categorical variables were compared using the chi-square or Fisher’s exact test, as appropriate. Binary logistic regression was applied to identify independent predictors of malignancy. Diagnostic efficacy was evaluated by calculating the AUROC, and differences between AUCs were tested using the DeLong test. P-value < 0.05 was considered statistically significant.

## 3 Results

### 3.1 Pathological classification and corresponding ultrasonographic features

As anticipated, benign and malignant NML exhibited distinct sonographic characteristics. Benign lesions, such as fibroadenomas and intraductal papillomas, were more frequently associated with multiple clustered cystic structures and hypoechoic areas with ill-defined margins. In contrast, malignant lesions, such as ductal carcinoma *in situ* (DCIS), invasive ductal carcinoma (IDC), and invasive lobular carcinoma (ILC), were more commonly characterized by hypoechoic areas with indistinct borders and architectural distortion. The distribution of pathological subtypes and their corresponding ultrasonographic features are summarized in (**Table 1**).

**Table 1:** Distribution of ultrasound features of non-mass-like breast lesions across different pathologic types.

Pathologic type	Clustered cystic areas	Ill-defined hypoechoic area	Architectural distortion	Posterior acoustic shadowing
Fibroadenoma (n=28)	11 (39.29%)	9 (32.14%)	1 (3.57%)	7 (25.00%)
Ductal Ectasia/Hyperplasia (n=27)	4 (14.81%)	7 (25.93%)	5 (18.52%)	11 (40.74%)
Intraductal Papilloma (n=17)	9 (52.94%)	4 (23.53%)	2 (11.76%)	2 (11.76%)
Inflammatory Disease (n=17)	7 (41.18%)	6 (35.29%)	3 (17.65%)	1 (5.88%)
Simple Hyperplasia (n=15)	4 (26.67%)	7 (46.67%)	1 (6.67%)	3 (20.00%)
Sclerosing Adenosis (n=7)	0	4 (57.14%)	2 (28.57%)	1 (14.29%)
Hamartoma (n=1)	0	1 (100%)	0	0
Benign lobular tumor (n=1)	0	1 (100%)	0	0
Atypical Ductal Hyperplasia (n=1)	1 (100.00%)	0	0	0
Papilloma + Sclerosing Adenosis (n=1)	0	0	1 (100%)	0
DCIS (n=40)	15 (37.50%)	18 (45.00%)	5 (12.50%)	2 (5.00%)
IDC (n=37)	8 (21.62%)	16 (43.24%)	7 (18.92%)	6 (16.22%)
DCIS + IDC (n=28)	5 (17.86%)	9 (32.14%)	12 (42.86%)	2 (7.14%)
ILC (n=9)	0	2 (22.22%)	4 (44.44%)	3 (33.33%)
DCIS + ILC (n=3)	1 (33.33%)	2 (66.67%)	0	0
Solid Papillary Carcinoma (n=3)	1 (33.33%)	2 (66.67%)	0	0
IDC + Mucinous Carcinoma (n=2)	1 (50.00%)	0	0	1 (50.00%)
Encapsulated Papillary Carcinoma (n=1)	1 (100.00%)	0	0	0
DCIS + Papilloma (n=1)	0	0	1 (100.00%)	0
DCIS + IDC + ILC (n=1)	0	0	1 (100.00%)	0

### 3.2 Clinical variables

Patients aged 45 years and older, as well as those presenting with palpable masses, were significantly more likely to exhibit malignant lesions compared to younger patients and those without palpable masses. Other clinical factors, including family history of breast cancer, breast pain, and nipple discharge, were not significantly associated with malignancy in this cohort (*P*>0.05) (**Table 2**).

**Table 2:** Univariate analysis of clinical characteristics associated with benign and malignant non-mass-like breast lesions.

Characteristic	Category	No. of benign lesions (n = 115)	No. of malignant lesions (n = 125)	Odds ratio (95% CI^2^)	*P* value
Age	<45 y	61 (53.04%)	30 (24.00%)	3.58	<0.001
	≥45 y	54 (49.96%)	95 (76.00%)	—	
Family History of carcinoma^1^	No	114 (99.13%)	121 (96.80%)	0.66	0.418
	Yes	1 (0.87%)	4 (3.20%)	—	
Breast Pain	No	84 (73.04%)	96 (76.80%)	0.27	0.783
	Yes	31 (26.96%)	29 (23.20%)	—	
Nipple Discharge	No	105 (91.30%)	106 (84.80%)	2.39	0.122
	Yes	10 (8.70%)	19 (15.20%)	—	
Palpable Lesion	No	57 (49.57%)	32 (25.60%)	14.74	<0.001
	Yes	58 (50.43%)	93 (74.40%)	—	

^1^Family History of Carcinoma was defined as the presence of breast cancer in a first-degree relative (mother, sister, or daughter).

^2^CI, confidence interval.

### 3.3 Ultrasonographic variables

Significant differences were observed between benign and malignant NML regarding several sonographic features, including maximum lesion diameter ≥1.5 cm, presence of internal vascularity, architectural distortion, intralesional microcalcifications, and abnormal axillary lymph nodes (*P* < 0.05) (**Table 3**).

**Table 3:** Univariate analysis of US characteristics associated with benign and malignant non-mass-like breast lesions.

Characteristic	Category	No. of benign lesions (n = 115)	No. of malignant lesions (n = 125)	chi-square value	*P* Value
Lesion Location	Right	57 (49.57%)	62 (49.60%)	0.00	0.996
	Left	58 (50.43%)	63 (50.40%)	—	
Maximum Lesion Diameter	<1.5 cm	58 (50.43%)	28 (22.40%)	20.47	<0.001
	≧1.5cm	57 (49.57%)	97 (77.60%)	—	
CDFI	No	70 (60.87%)	35 (28.00%)	26.30	<0.001
	Yes	45 (39.13%)	90 (72.00%)	—	
Microcalcifications^1^	No	74 (64.35%)	39 (31.20%)	26.42	<0.001
	Yes	41 (35.65%)	86 (68.8%)	—	
Architectural Distortion	No	85 (73.91%)	73 (58.40%)	5.00	0.025
	Yes	30 (26.09%)	52 (41.60%)	—	
Posterior Acoustic Shadowing	No	90 (78.26%)	108 (86.40%)	2.75	0.097
	Yes	25 (21.74%)	17 (13.60%)	—	
Abnormal Axillary Lymph Nodes	No	112 (97.39%)	103 (82.40%)	14.43	<0.001
	Yes	3 (2.61%)	22 (17.60%)	—	
	3	8 (6.96%)	4 (3.20%)	—	—
	4A	20 (17.39%)	15 (12.00%)		
BI-RADS	4B	9 (7.83%)	28 (22.40%)		
	4C	6 (5.22%)	38 (30.40%)		
	5	0 (0%)	5 (4.00%)		

^1^Microcalcifications on ultrasound were defined as punctate hyperechoic foci. These findings were labeled as “microcalcifications” for consistency, regardless of visibility on digital mammography.

**Figure 3:**
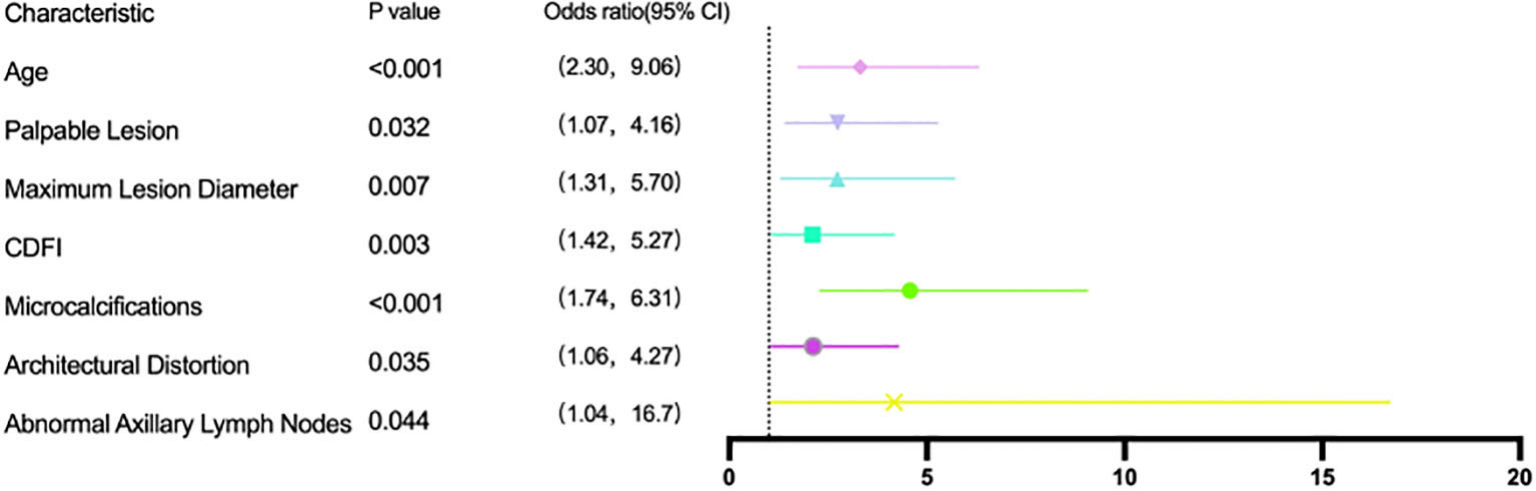
Multivariate logistic regression of clinical and ultrasonographic predictors of NML malignancy.

Multifactorial logistic regression analysis was conducted for ultrasound and clinical signs that displayed significance in one-way tests, all of which remained statistically significant associations with malignancy (*P* < 0.05) (**Figure 3**).

### 3.4 Diagnostic efficacy

#### 3.4.1 Diagnostic efficacy of individual imaging modalities

**Figure 4:**
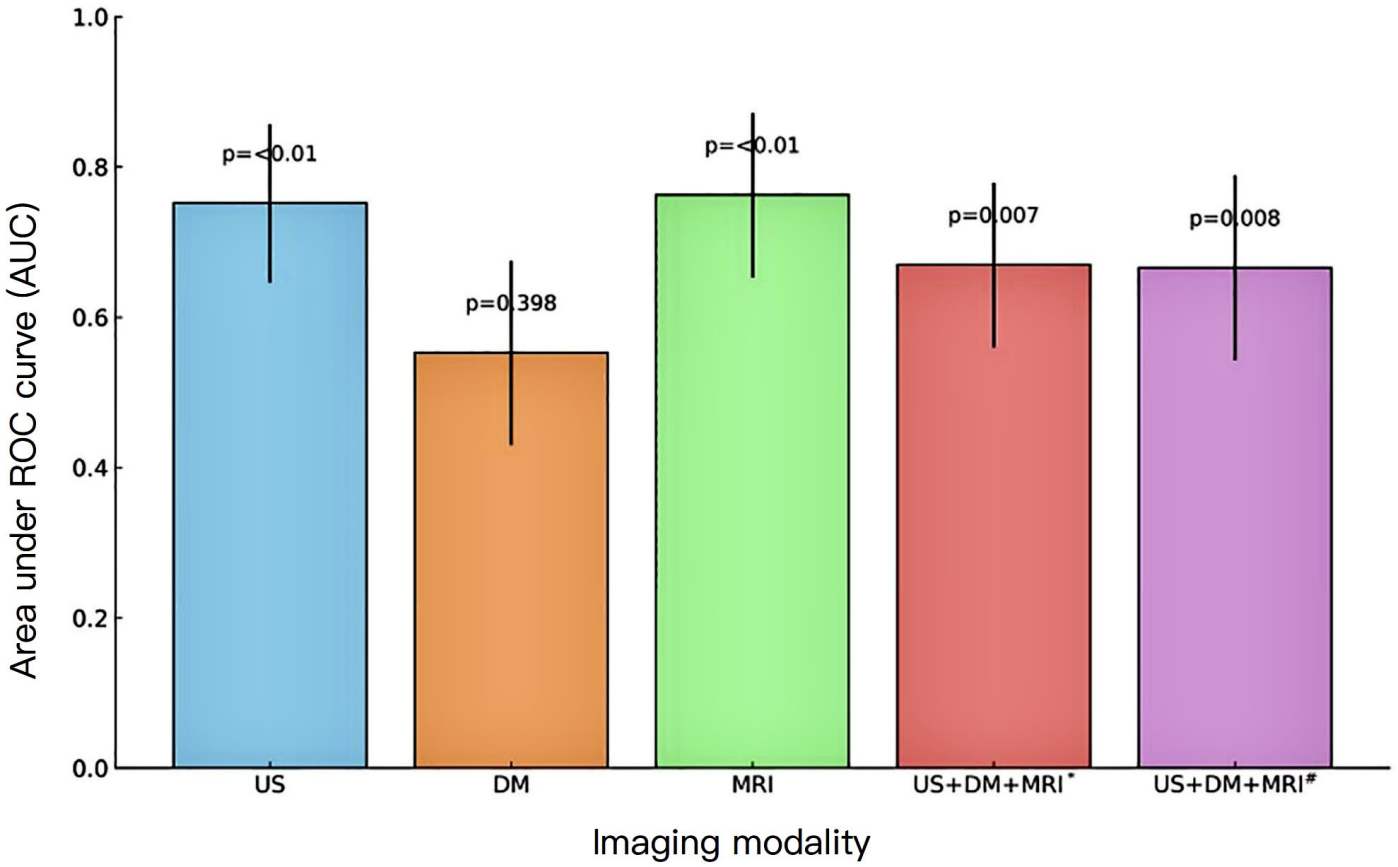
Comparison of AUC values and 95% confidence intervals for diagnostic imaging strategies. *Tandem combination: all three imaging modalities must be positive to yield a positive diagnosis. ^#^Parallel combination: a positive result from any one of the three modalities is considered positive.

Ultrasonography identified 240 NMLs in 235 patients, including 115 benign (47.9%) and 125 malignant (52.1%) lesions. Among these patients, 155 patients underwent DM, which revealed 157 NMLs (81 benign [51.6%] and 76 malignant [48.4%]), while 130 patients underwent DCE-MRI, which identified 133 NMLs (37 benign [27.8%] and 96 malignant [72.2%]). Diagnostic performance metrics were calculated for each modality and visualized using radar charts (**Figure 4**).

Between-group comparisons uncovered significant differences between US, DM, and DCE-MRI in terms of accuracy, sensitivity and PPV (All *P* < 0.05). However, a borderline significance was noted for specificity (*P* = 0.0458), while NPV was comparable (*P* = 0.192) (**Table 4**).

**Table 4:** Diagnostic performance metrics of breast imaging modalities and between-group comparisons.

Imaging modality	Diagnostic performance(%)				
	Accuracy	Sensitivity	Specificity	PPV	NPV
US	80.42	80.80	80.00	81.45	79.31
DM	66.88	65.79	67.90	65.79	67.90
DCE-MRI	80.45	88.89	62.79	83.33	72.97
*P*	0.0036	0.0106	0.0458	0.0107	0.192

Interestingly, DCE-MRI displayed the highest sensitivity (88.89%) and PPV (83.33%) and a relatively higher accuracy (80.45%). US exhibited balanced performance across all five metrics (80.42% accuracy, 80.80% sensitivity, 80.00% specificity, 81.45% PPV, and 79.31% NPV), reflecting its stable diagnostic capability. In contrast, DM yielded the lowest overall performance, with an accuracy and sensitivity of 66.88% and 65.79%, respectively, indicating a significantly lower diagnostic performance than US and DCE-MRI.

#### 3.4.2 Evaluation of multimodal imaging performance

**Figure 5:**
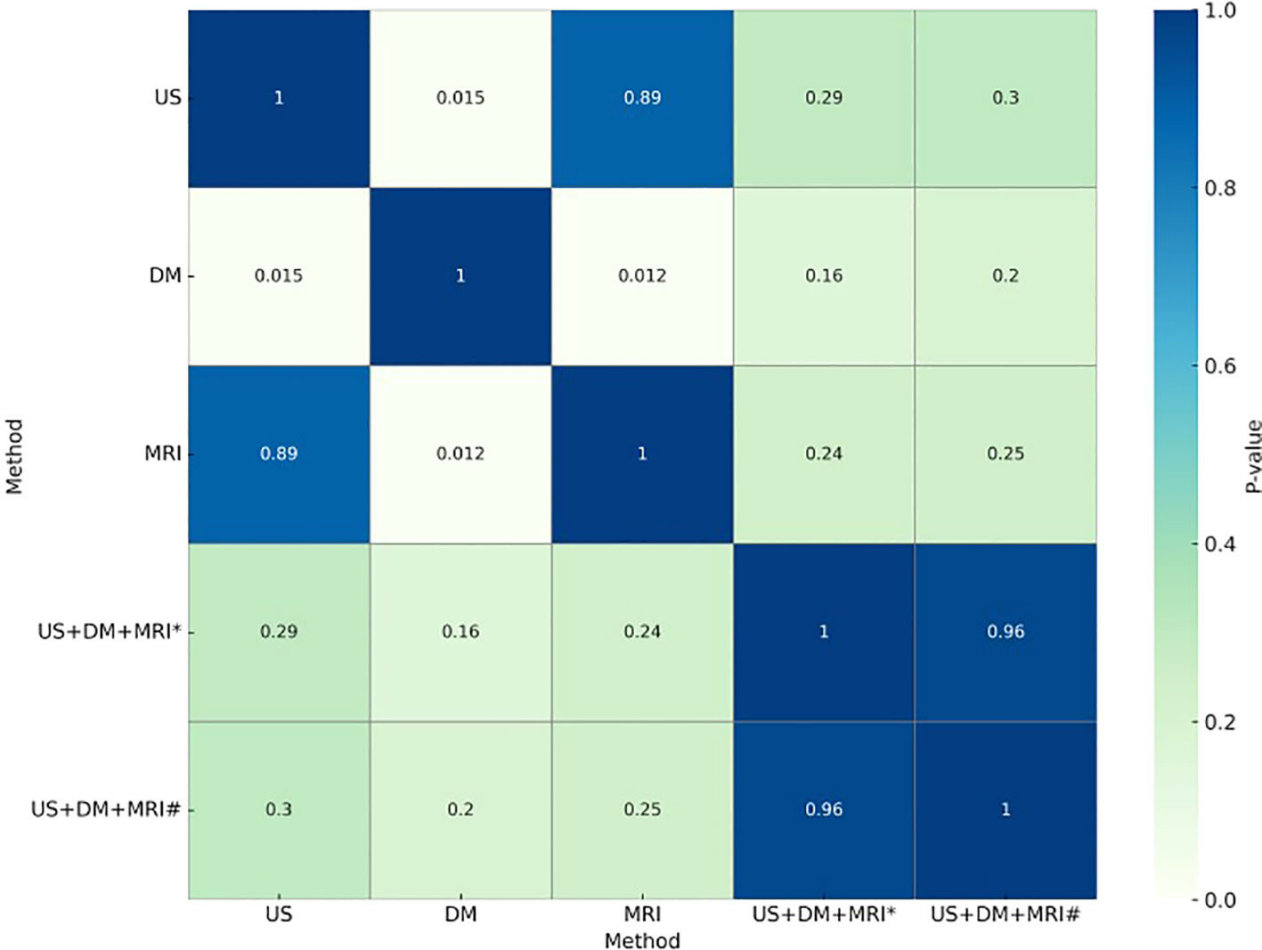
Pairwise comparison of AUC values using the delong test. *Tandem combination: all three imaging modalities must be positive to yield a positive diagnosis. ^#^Parallel combination: a positive result from any one of the three modalities is considered positive.

A total of 95 patients undergoing all three imaging modalities were included, with 96 NMLs identified (33 benign [34.4%], 63 malignant [65.6%]). Comparative analysis was performed across five diagnostic metrics: accuracy, sensitivity, specificity, PPV, and NPV (**Figures 4**, **5**).

AUC analysis demonstrated significant differences between ultrasound and DM (*P* < 0.001) and between DM and MRI (*P* < 0.01), while ultrasound and MRI showed no difference (*P*=0.856), suggesting comparable overall diagnostic performance. Combined diagnostic approaches did not yield statistically significant AUC improvements over individual modalities (*P*≥0.05).

## 4 Discussion

Herein, while DCE-MRI displayed outstanding sensitivity (88.89%) and PPV (83.33%), its specificity was merely 62.79%, which was lower than that of US (80.00%) suggesting that it may be associated with an increased risk of false positives and improved detection rates. On the other hand, the performance of US was more balanced in terms of accuracy (80.42%), sensitivity (80.80%), specificity (80.00%) and PPV (81.45%) and NPV (79.31%). In contrast, the five diagnostic efficacy indicators of DM were significantly lower than those of US and MRI, with an accuracy of 66.88%, a sensitivity and PPV of 65.79%, and a specificity of 67.90%. This result suggests that the overall limited efficacy of DM in NML detection may be related to its ability to visualize low-density lesions in non-calcified lesions. While the diagnostic performance of combined modalities, both in tandem and parallel configurations, improved some indexes, the results of the AUC comparison revealed no significant advantage over single-modality assessments in terms of overall diagnostic efficacy (*P*>0.05), signaling that the combination of the multimodal modalities does not necessarily substantially enhance diagnostic efficacy.

In conclusion, ultrasound is recommended as the preferred screening tool for evaluating non-mass-like breast lesions owing to its high sensitivity and specificity. Additionally, DCE-MRI can be utilized for further assessment of ultrasound-suggested high-risk lesions, particularly when lesions are located in the deeper areas of the breast or when their borders are poorly defined, given that MRI provides superior tissue contrast. Although DM demonstrates lower overall efficacy, it remains essential for detecting microcalcifications and is recommended as a complementary tool to ultrasound for optimizing diagnostic accuracy (17–19).

The pathologic types of NML in the present study included benign, malignant, and junctional lesions, with DCIS identified as the primary subtype of malignant NML, a finding consistent with that of previous studies (20, 21). Besides, the combination of DCIS with invasive carcinoma (e.g., IDC or ILC) significantly increased the incidence of structural distortion, indicating the infiltrative and destructive effect of invasive carcinoma on the surrounding tissue structure (22). In addition to the common structural distortions, posterior acoustic shadows were also detected in cases of IDC alone, suggesting that the lesion is accompanied by significant interstitial fibrosis and tissue sclerosis (22). Of note, some sonographic differences were noted between different malignant subtypes. For instance, DCIS patients generally exhibited microcalcifications, ductal arrangement, and structural preservation, with fewer “destructive” imaging signs, whereas IDC was more infiltrative, with marked structural distortions, echogenicity, and posterior acoustic shadows, implying a more pronounced destructive effect on adjacent tissue structures (23).

Conversely, ascribed to the loose arrangement of ILC cells, the insidious nature of interstitial infiltration, and the lack of distinct mass-like structures, ILCs commonly exhibited nonspecific hypoechoic areas or mild structural abnormalities on ultrasound, thereby complicating clinical detection. According to earlier research, structural distortion may be indicative of lesion infiltration (however, relying on this sign necessitates the exclusion of postoperative scarring in the area), while vague hypoechoic borders or clustered cyst-like structures were more frequently observed in benign or *in situ* lesions (10). Herein, the sample size of encapsulated papillary carcinoma and solid papillary carcinoma *in situ* was limited; thus, the diagnosis should be further confirmed by combining immunohistochemical pathological findings and immunohistochemical markers. The sonographic manifestations of NML are closely related to its pathological type, highlighting the importance of integrating both imaging and pathological findings in clinical decision-making.

It is worthwhile emphasizing that the 5th edition of the BI-RADS classification system has not yet formally incorporated NML into its standardized classification, leading to ambiguity in image interpretation and risk stratification (24). Nevertheless, attempts have been made to evaluate the applicability of the current ultrasound BI-RADS classification in assessing NML, but its diagnostic efficacy is limited compared to mass-type lesions (25). Although the ultrasound BI-RADS classification showed superior sensitivity (80.80%) and specificity (80.00%) to DM (65.79%, 67.90%) in this study, it is limited by its insufficient adaptability and subjectivity in the application of NMLs. We posit that the sixth edition of the BI-RADS system will be updated to formally incorporate the definition of NML and grading recommendations.

Noteworthily, current ultrasound classification criteria for NML are not standardized. The Japanese Association of Breast and Thyroid Ultrasound (JABTS) classifies NML into five categories (13), namely mammary hypoechoic areas, ductal abnormalities, structural distortions, multiple small cysts, and strongly echogenic foci without a hypoechoic background. Ko et al. (26), on the other hand, proposed a four-type classification method that incorporates morphology, calcification, and sonographic characteristics, consisting of ductal hypoechoic areas (Ia/Ib), nonductal hypoechoic areas (IIa/IIb), structural distortions (III), and lesions with posterior acoustic shadows (IV). Importantly, both types Ib and IIb are associated with microcalcifications, which are strongly associated with high-risk lesions. However, it has also been pointed out that it is challenging to distinguish between ductal and non-ductal hypoechoic areas on two-dimensional images in the clinical setting (24). Thus, we propose to simplify and categorize sonographic subtypes as follows: multiple clustered cystic lesions, hypoechoic areas with indistinct borders, structural distortion, and lesions accompanied by posterior acoustic shadows.

The descriptions used in the BI-RADS system for malignant masses (e.g., ill-defined borders, abundant blood flow, calcification) are also applicable to the determination of malignant NML. In this study, the BI-RADS classification and the NML classification proposed by Ko et al. were combined to further identify key features with predictive value in NML. The detection rate of microcalcifications in this study was 52.9%, in line with those reported in previous studies (27, 28) and highlighting its clinical value in identifying carcinoma *in situ*, such as DCIS. Structural distortion was also identified as a significant predictor (*P* < 0.05), despite being challenging to recognize in two-dimensional images. However, it can be more effectively captured by Ultrasound examination in real-time (29, 30). Meanwhile, CDFI is also valuable in the assessment of blood flow patterns in NML, with several studies concluding that malignant NMLs are more likely to present with abundant blood flow, which can assist in the differentiation between benign and malignant lesions (8, 9). Therefore, it is recommended to combine multi-parametric ultrasonographic features for the evaluation of NML to improve diagnostic reliability and to provide a reference for subsequent optimization of the BI-RADS classification.

This study has several limitations. First, as a retrospective single-center study, the sample source was relatively limited, which may have introduced selection bias and consequently restricted the generalizability of the findings. Second, the similarity of imaging features and diagnostic results may have been impacted by differences in the timing and operators of imaging exams. Third, there was little statistical power due to the small number of cases for some pathological categories (such as solid papillary carcinoma *in situ* and encapsulated papillary carcinoma). Finally, the absence of multicenter, prospective validation and external datasets limits the robustness and external applicability of the proposed sonographic feature classification method. Therefore, more multicenter, large-scale prospective multicenter studies are required to confirm the clinical value of this approach.

## 5 Conclusion

In conclusion, this study indicated that digital mammography, ultrasonography, and DCE-MRI each have unique benefits when it comes to diagnosing non-mass-like breast lesions. DCE-MRI provides more detailed information for characterizing lesions, while ultrasound is more useful in clinical settings and has a higher diagnostic specificity. Despite its limitations in detecting non-calcified lesions, digital mammography is nevertheless a viable supplementary technique for detecting microcalcifications. Combining these imaging modalities can enhance diagnostic precision and enable more comprehensive clinical decision-making. More multicenter, extensive, and prospective research is necessary to confirm these results and develop standardized, multimodal diagnostic models for non-mass-like breast lesions.

## Data Availability

The raw data supporting the conclusions of this article will be made available by the authors, without undue reservation.
